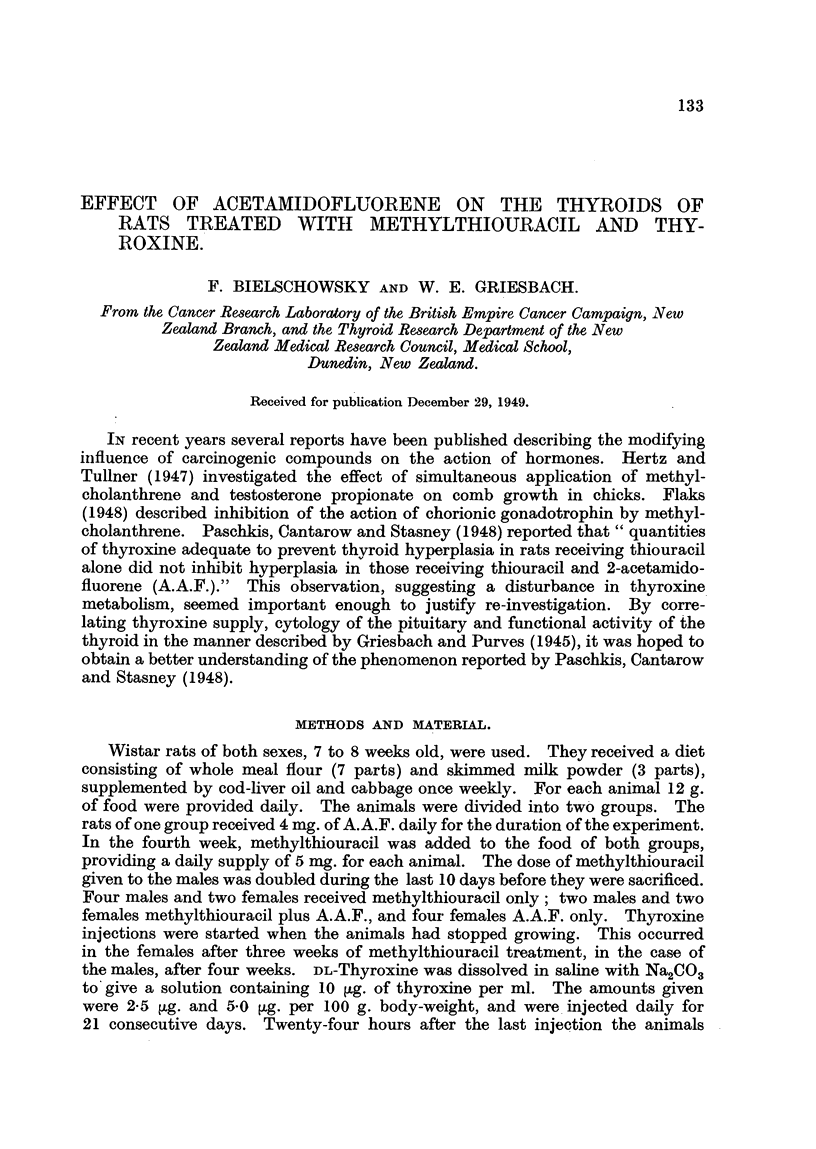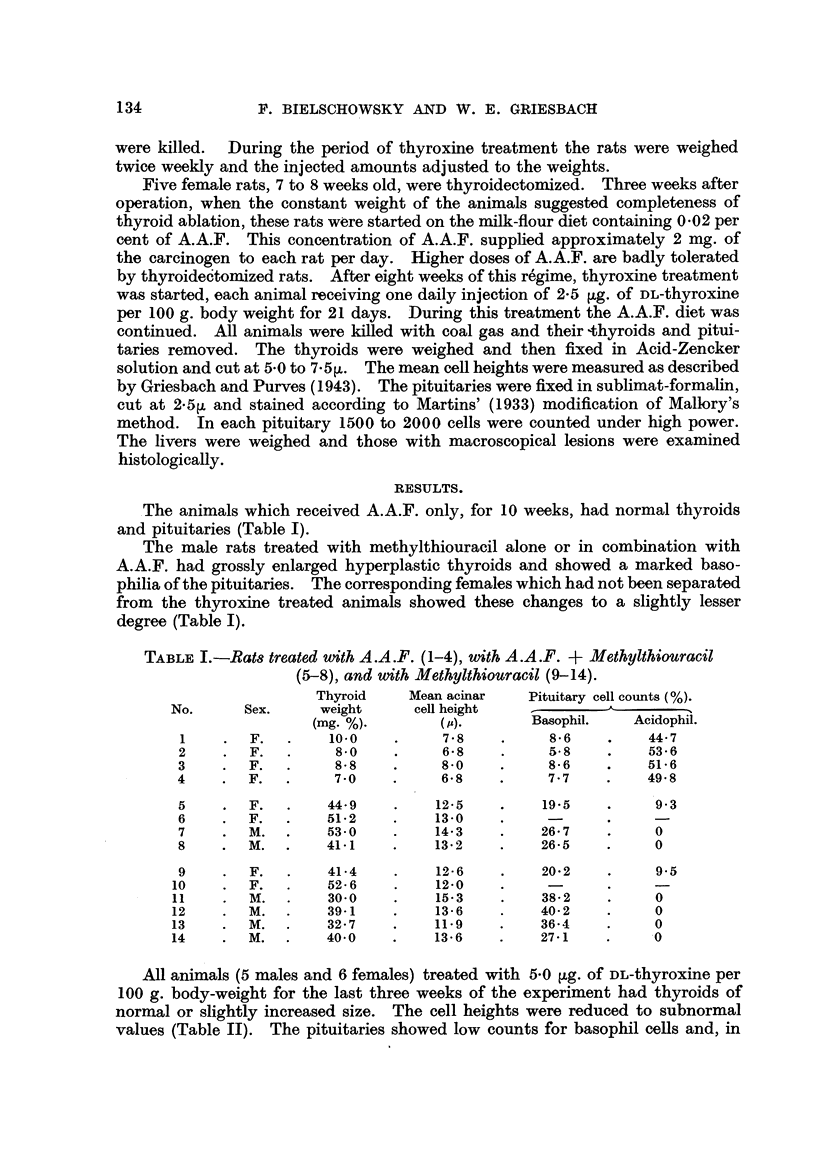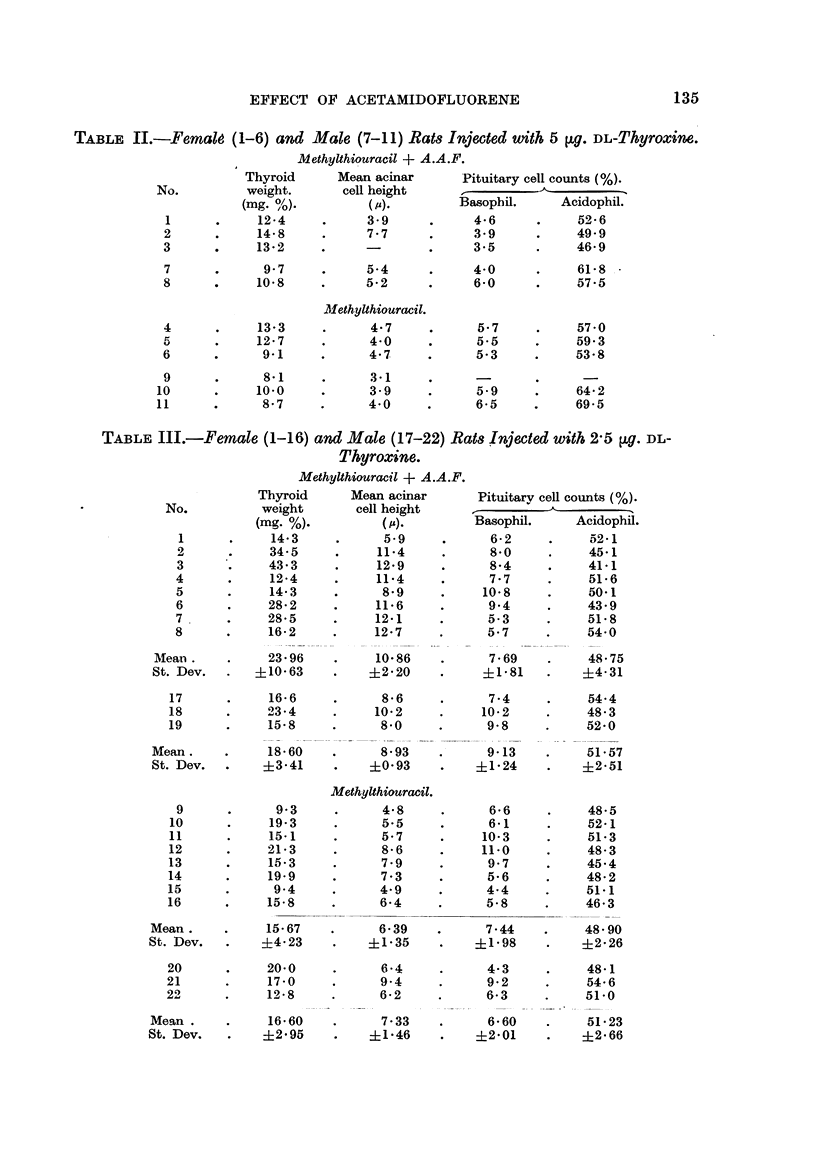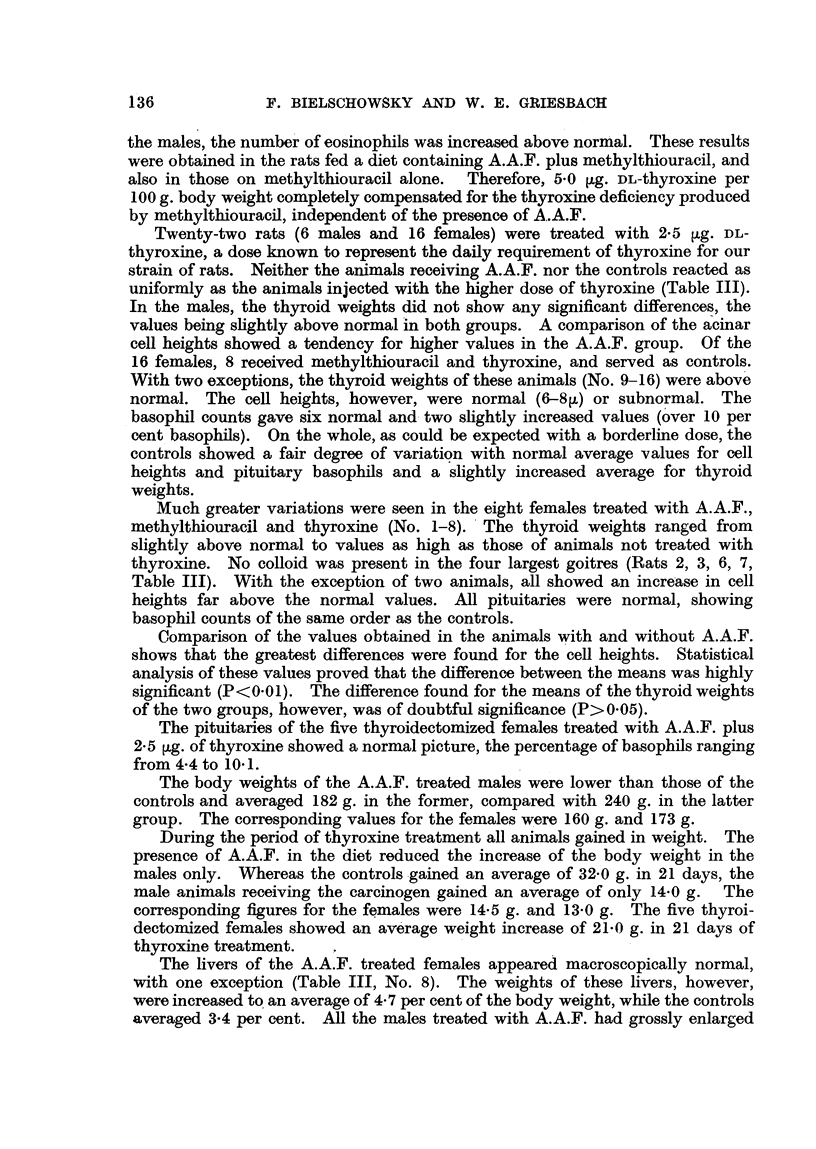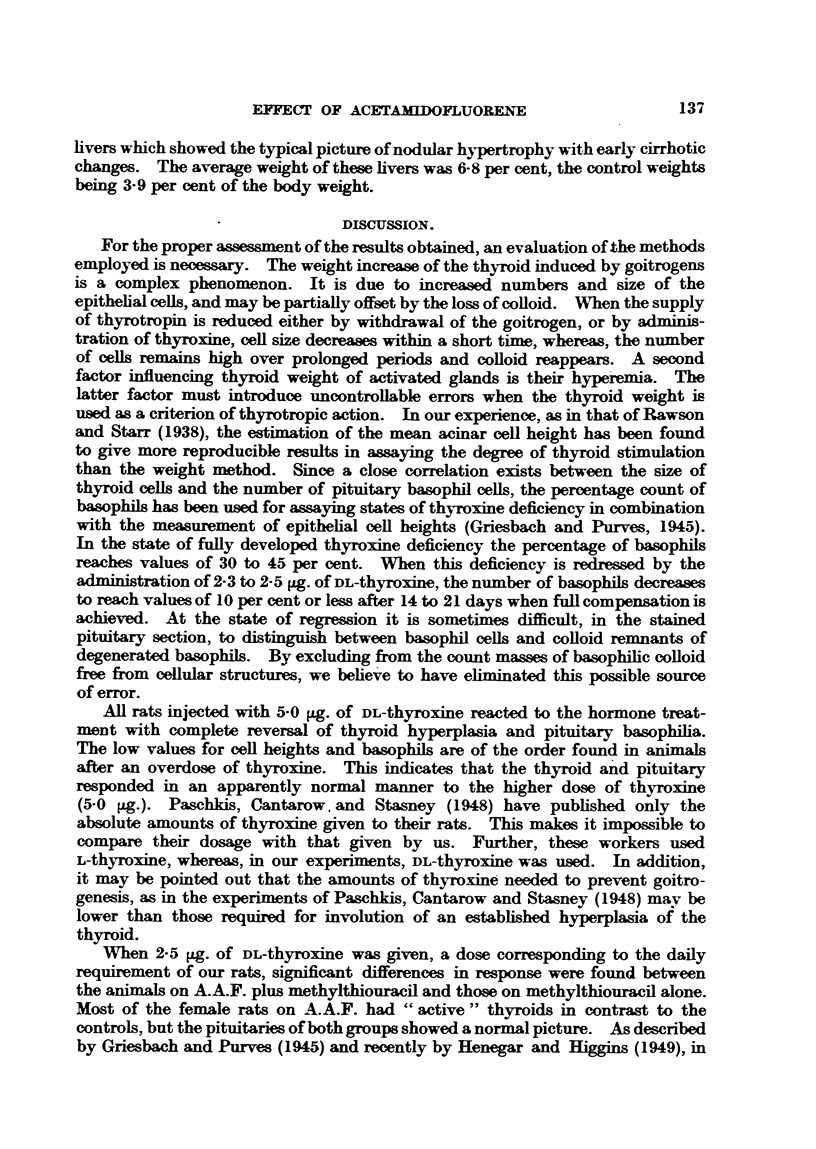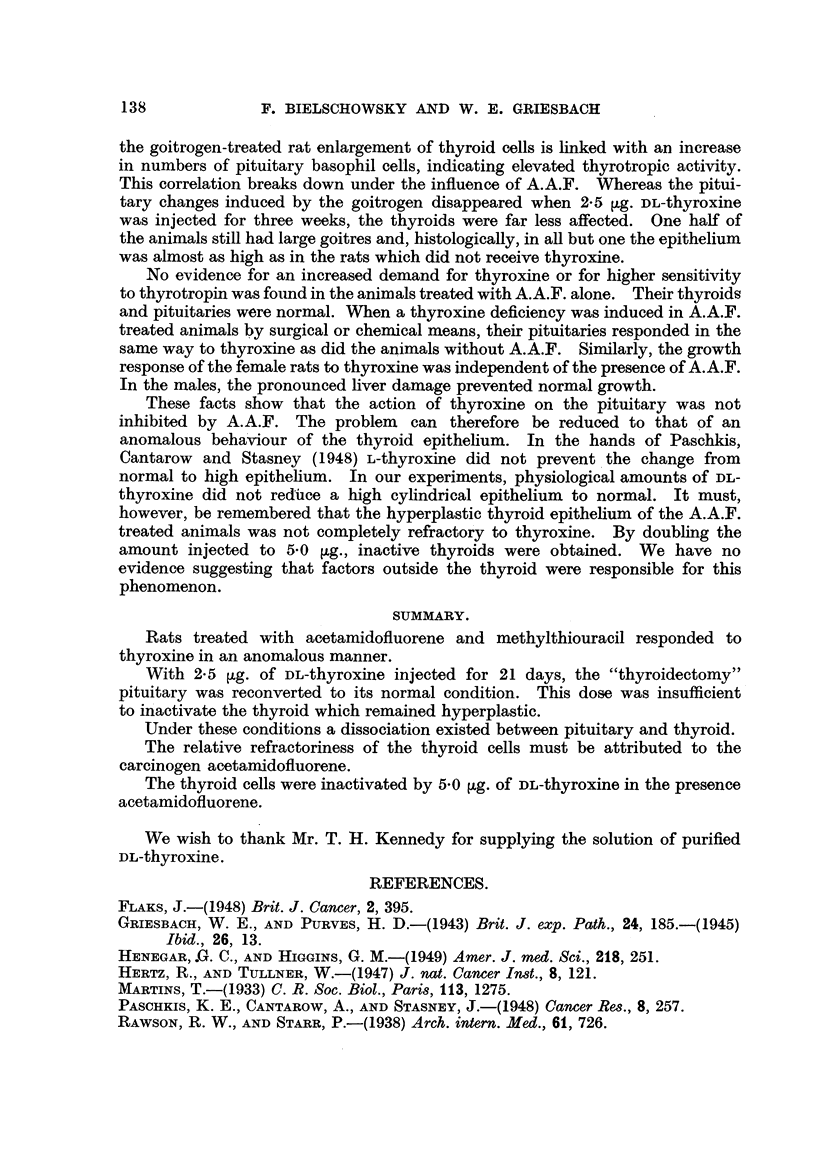# Effect of Acetamidofluorene on the Thyroids of Rats treated with Methylthiouracil and Thyroxine

**DOI:** 10.1038/bjc.1950.13

**Published:** 1950-03

**Authors:** F. Bielschowsky, W. E. Griesbach


### EFFECT OF ACETAMIDOFLUORENE ON THE THYROIDS OF RATS TREATED WITH METHYLTHIOURACIL AND THYROXINE.

F. BIELSCHOWSKY AND W. E. GRIESBACH.

*From the Cancer Research Laboratory of the British Empire Cancer Campaign, New Zealand Branch, and the Thyroid Research Department of the New Zealand Medical Research Council, Medical School, Dunedin, New Zealand.*

Received for publication December 29, 1949.

In recent years several reports have been published describing the modifying influence of carcinogenic compounds on the action of hormones. Hertz and Tullner (1947) investigated the effect of simultaneous application of methylcholanthrene and testosterone propionate on comb growth in chicks. Flaks (1948) described inhibition of the action of chorionic gonadotrophin by methylcholanthrene. Paschkis, Cantarow and Stasney (1948) reported that " quantities of thyroxine adequate to prevent thyroid hyperplasia in rats receiving thiouracil alone did not inhibit hyperplasia in those receiving thiouracil and 2-acetamidofluorene (A.A.F.)." This observation, suggesting a disturbance in thyroxine metabolism, seemed important enough to justify re-investigation. By correlating thyroxine supply, cytology of the pituitary and functional activity of the thyroid in the manner described by Griesbach and Purves (1945), it was hoped to obtain a better understanding of the phenomenon reported by Paschkis, Cantarow and Stasney (1948).

### METHODS AND MATERIAL.

Wistar rats of both sexes, 7 to 8 weeks old, were used. They received a diet consisting of whole meal flour (7 parts) and skimmed milk powder (3 parts), supplemented by cod-liver oil and cabbage once weekly. For each animal 12 g. of food were provided daily. The animals were divided into two groups. The rats of one group received 4 mg. of A.A.F. daily for the duration of the experiment. In the fourth week, methylthiouracil was added to the food of both groups, providing a daily supply of 5 mg. for each animal. The dose of methylthiouracil given to the males was doubled during the last 10 days before they were sacrificed. Four males and two females received methylthiouracil only ; two males and two females methylthiouracil plus A.A.F., and four females A.A.F. only. Thyroxine injections were started when the animals had stopped growing. This occurred in the females after three weeks of methylthiouracil treatment, in the case of the males, after four weeks. DL-Thyroxine was dissolved in saline with $Na_2CO_3$ to give a solution containing 10 μg. of thyroxine per ml. The amounts given were 2·5 μg. and 5·0 μg. per 100 g. body-weight, and were injected daily for 21 consecutive days. Twenty-four hours after the last injection the animals were killed. During the period of thyroxine treatment the rats were weighed twice weekly and the injected amounts adjusted to the weights.

Five female rats, 7 to 8 weeks old, were thyroidectomized. Three weeks after operation, when the constant weight of the animals suggested completeness of thyroid ablation, these rats were started on the milk-flour diet containing 0·02 per cent of A.A.F. This concentration of A.A.F. supplied approximately 2 mg. of the carcinogen to each rat per day. Higher doses of A.A.F. are badly tolerated by thyroidectomized rats. After eight weeks of this régime, thyroxine treatment was started, each animal receiving one daily injection of 2·5 μg. of DL-thyroxine per 100 g. body weight for 21 days. During this treatment the A.A.F. diet was continued. All animals were killed with coal gas and their thyroids and pituitaries removed. The thyroids were weighed and then fixed in Acid-Zencker solution and cut at 5·0 to 7·5μ. The mean cell heights were measured as described by Griesbach and Purves (1943). The pituitaries were fixed in sublimat-formalin, cut at 2·5μ and stained according to Martins' (1933) modification of Mallory's method. In each pituitary 1500 to 2000 cells were counted under high power. The livers were weighed and those with macroscopical lesions were examined histologically.

### RESULTS.

The animals which received A.A.F. only, for 10 weeks, had normal thyroids and pituitaries (Table I).

The male rats treated with methylthiouracil alone or in combination with A.A.F. had grossly enlarged hyperplastic thyroids and showed a marked basophilia of the pituitaries. The corresponding females which had not been separated from the thyroxine treated animals showed these changes to a slightly lesser degree (Table I).

TABLE I.—*Rats treated with A.A.F.* (1–4), *with A.A.F. + Methylthiouracil* (5–8), *and with Methylthiouracil* (9–14).

| No. | Sex. | Thyroid weight (mg. %). | Mean acinar cell height (μ). | Pituitary cell counts (%). Basophil. | Acidophil. |
|---|---|---|---|---|---|
| 1 | F. | 10·0 | 7·8 | 8·6 | 44·7 |
| 2 | F. | 8·0 | 6·8 | 5·8 | 53·6 |
| 3 | F. | 8·8 | 8·0 | 8·6 | 51·6 |
| 4 | F. | 7·0 | 6·8 | 7·7 | 49·8 |
| 5 | F. | 44·9 | 12·5 | 19·5 | 9·3 |
| 6 | F. | 51·2 | 13·0 | — | — |
| 7 | M. | 53·0 | 14·3 | 26·7 | 0 |
| 8 | M. | 41·1 | 13·2 | 26·5 | 0 |
| 9 | F. | 41·4 | 12·6 | 20·2 | 9·5 |
| 10 | F. | 52·6 | 12·0 | — | — |
| 11 | M. | 30·0 | 15·3 | 38·2 | 0 |
| 12 | M. | 39·1 | 13·6 | 40·2 | 0 |
| 13 | M. | 32·7 | 11·9 | 36·4 | 0 |
| 14 | M. | 40·0 | 13·6 | 27·1 | 0 |

All animals (5 males and 6 females) treated with 5·0 μg. of DL-thyroxine per 100 g. body-weight for the last three weeks of the experiment had thyroids of normal or slightly increased size. The cell heights were reduced to subnormal values (Table II). The pituitaries showed low counts for basophil cells and, in

TABLE II.—*Female (1–6) and Male (7–11) Rats Injected with 5 μg. DL-Thyroxine.*

| No. | Thyroid weight. (mg. %). | Mean acinar cell height (μ). | Pituitary cell counts (%). Basophil. | Acidophil. |
|---|---|---|---|---|
| | | *Methylthiouracil + A.A.F.* | | |
| 1 | 12·4 | 3·9 | 4·6 | 52·6 |
| 2 | 14·8 | 7·7 | 3·9 | 49·9 |
| 3 | 13·2 | — | 3·5 | 46·9 |
| 7 | 9·7 | 5·4 | 4·0 | 61·8 |
| 8 | 10·8 | 5·2 | 6·0 | 57·5 |
| | | *Methylthiouracil.* | | |
| 4 | 13·3 | 4·7 | 5·7 | 57·0 |
| 5 | 12·7 | 4·0 | 5·5 | 59·3 |
| 6 | 9·1 | 4·7 | 5·3 | 53·8 |
| 9 | 8·1 | 3·1 | — | — |
| 10 | 10·0 | 3·9 | 5·9 | 64·2 |
| 11 | 8·7 | 4·0 | 6·5 | 69·5 |

TABLE III.—*Female (1–16) and Male (17–22) Rats Injected with 2·5 μg. DL-Thyroxine.*

| No. | Thyroid weight (mg. %). | Mean acinar cell height (μ). | Pituitary cell counts (%). Basophil. | Acidophil. |
|---|---|---|---|---|
| | | *Methylthiouracil + A.A.F.* | | |
| 1 | 14·3 | 5·9 | 6·2 | 52·1 |
| 2 | 34·5 | 11·4 | 8·0 | 45·1 |
| 3 | 43·3 | 12·9 | 8·4 | 41·1 |
| 4 | 12·4 | 11·4 | 7·7 | 51·6 |
| 5 | 14·3 | 8·9 | 10·8 | 50·1 |
| 6 | 28·2 | 11·6 | 9·4 | 43·9 |
| 7 | 28·5 | 12·1 | 5·3 | 51·8 |
| 8 | 16·2 | 12·7 | 5·7 | 54·0 |
| Mean | 23·96 | 10·86 | 7·69 | 48·75 |
| St. Dev. | ±10·63 | ±2·20 | ±1·81 | ±4·31 |
| 17 | 16·6 | 8·6 | 7·4 | 54·4 |
| 18 | 23·4 | 10·2 | 10·2 | 48·3 |
| 19 | 15·8 | 8·0 | 9·8 | 52·0 |
| Mean | 18·60 | 8·93 | 9·13 | 51·57 |
| St. Dev. | ±3·41 | ±0·93 | ±1·24 | ±2·51 |
| | | *Methylthiouracil.* | | |
| 9 | 9·3 | 4·8 | 6·6 | 48·5 |
| 10 | 19·3 | 5·5 | 6·1 | 52·1 |
| 11 | 15·1 | 5·7 | 10·3 | 51·3 |
| 12 | 21·3 | 8·6 | 11·0 | 48·3 |
| 13 | 15·3 | 7·9 | 9·7 | 45·4 |
| 14 | 19·9 | 7·3 | 5·6 | 48·2 |
| 15 | 9·4 | 4·9 | 4·4 | 51·1 |
| 16 | 15·8 | 6·4 | 5·8 | 46·3 |
| Mean | 15·67 | 6·39 | 7·44 | 48·90 |
| St. Dev. | ±4·23 | ±1·35 | ±1·98 | ±2·26 |
| 20 | 20·0 | 6·4 | 4·3 | 48·1 |
| 21 | 17·0 | 9·4 | 9·2 | 54·6 |
| 22 | 12·8 | 6·2 | 6·3 | 51·0 |
| Mean | 16·60 | 7·33 | 6·60 | 51·23 |
| St. Dev. | ±2·95 | ±1·46 | ±2·01 | ±2·66 |

the males, the number of eosinophils was increased above normal. These results were obtained in the rats fed a diet containing A.A.F. plus methylthiouracil, and also in those on methylthiouracil alone. Therefore, 5·0 μg. DL-thyroxine per 100 g. body weight completely compensated for the thyroxine deficiency produced by methylthiouracil, independent of the presence of A.A.F.

Twenty-two rats (6 males and 16 females) were treated with 2·5 μg. DL-thyroxine, a dose known to represent the daily requirement of thyroxine for our strain of rats. Neither the animals receiving A.A.F. nor the controls reacted as uniformly as the animals injected with the higher dose of thyroxine (Table III). In the males, the thyroid weights did not show any significant differences, the values being slightly above normal in both groups. A comparison of the acinar cell heights showed a tendency for higher values in the A.A.F. group. Of the 16 females, 8 received methylthiouracil and thyroxine, and served as controls. With two exceptions, the thyroid weights of these animals (No. 9–16) were above normal. The cell heights, however, were normal (6–8μ) or subnormal. The basophil counts gave six normal and two slightly increased values (over 10 per cent basophils). On the whole, as could be expected with a borderline dose, the controls showed a fair degree of variation with normal average values for cell heights and pituitary basophils and a slightly increased average for thyroid weights.

Much greater variations were seen in the eight females treated with A.A.F., methylthiouracil and thyroxine (No. 1–8). The thyroid weights ranged from slightly above normal to values as high as those of animals not treated with thyroxine. No colloid was present in the four largest goitres (Rats 2, 3, 6, 7, Table III). With the exception of two animals, all showed an increase in cell heights far above the normal values. All pituitaries were normal, showing basophil counts of the same order as the controls.

Comparison of the values obtained in the animals with and without A.A.F. shows that the greatest differences were found for the cell heights. Statistical analysis of these values proved that the difference between the means was highly significant ($P < 0{\cdot}01$). The difference found for the means of the thyroid weights of the two groups, however, was of doubtful significance ($P > 0{\cdot}05$).

The pituitaries of the five thyroidectomized females treated with A.A.F. plus 2·5 μg. of thyroxine showed a normal picture, the percentage of basophils ranging from 4·4 to 10·1.

The body weights of the A.A.F. treated males were lower than those of the controls and averaged 182 g. in the former, compared with 240 g. in the latter group. The corresponding values for the females were 160 g. and 173 g.

During the period of thyroxine treatment all animals gained in weight. The presence of A.A.F. in the diet reduced the increase of the body weight in the males only. Whereas the controls gained an average of 32·0 g. in 21 days, the male animals receiving the carcinogen gained an average of only 14·0 g. The corresponding figures for the females were 14·5 g. and 13·0 g. The five thyroidectomized females showed an average weight increase of 21·0 g. in 21 days of thyroxine treatment.

The livers of the A.A.F. treated females appeared macroscopically normal, with one exception (Table III, No. 8). The weights of these livers, however, were increased to an average of 4·7 per cent of the body weight, while the controls averaged 3·4 per cent. All the males treated with A.A.F. had grossly enlarged livers which showed the typical picture of nodular hypertrophy with early cirrhotic changes. The average weight of these livers was 6·8 per cent, the control weights being 3·9 per cent of the body weight.

### DISCUSSION.

For the proper assessment of the results obtained, an evaluation of the methods employed is necessary. The weight increase of the thyroid induced by goitrogens is a complex phenomenon. It is due to increased numbers and size of the epithelial cells, and may be partially offset by the loss of colloid. When the supply of thyrotropin is reduced either by withdrawal of the goitrogen, or by administration of thyroxine, cell size decreases within a short time, whereas, the number of cells remains high over prolonged periods and colloid reappears. A second factor influencing thyroid weight of activated glands is their hyperemia. The latter factor must introduce uncontrollable errors when the thyroid weight is used as a criterion of thyrotropic action. In our experience, as in that of Rawson and Starr (1938), the estimation of the mean acinar cell height has been found to give more reproducible results in assaying the degree of thyroid stimulation than the weight method. Since a close correlation exists between the size of thyroid cells and the number of pituitary basophil cells, the percentage count of basophils has been used for assaying states of thyroxine deficiency in combination with the measurement of epithelial cell heights (Griesbach and Purves, 1945). In the state of fully developed thyroxine deficiency the percentage of basophils reaches values of 30 to 45 per cent. When this deficiency is redressed by the administration of 2·3 to 2·5 μg. of DL-thyroxine, the number of basophils decreases to reach values of 10 per cent or less after 14 to 21 days when full compensation is achieved. At the state of regression it is sometimes difficult, in the stained pituitary section, to distinguish between basophil cells and colloid remnants of degenerated basophils. By excluding from the count masses of basophilic colloid free from cellular structures, we believe to have eliminated this possible source of error.

All rats injected with 5·0 μg. of DL-thyroxine reacted to the hormone treatment with complete reversal of thyroid hyperplasia and pituitary basophilia. The low values for cell heights and basophils are of the order found in animals after an overdose of thyroxine. This indicates that the thyroid and pituitary responded in an apparently normal manner to the higher dose of thyroxine (5·0 μg.). Paschkis, Cantarow and Stasney (1948) have published only the absolute amounts of thyroxine given to their rats. This makes it impossible to compare their dosage with that given by us. Further, these workers used L-thyroxine, whereas, in our experiments, DL-thyroxine was used. In addition, it may be pointed out that the amounts of thyroxine needed to prevent goitrogenesis, as in the experiments of Paschkis, Cantarow and Stasney (1948) may be lower than those required for involution of an established hyperplasia of the thyroid.

When 2·5 μg. of DL-thyroxine was given, a dose corresponding to the daily requirement of our rats, significant differences in response were found between the animals on A.A.F. plus methylthiouracil and those on methylthiouracil alone. Most of the female rats on A.A.F. had "active" thyroids in contrast to the controls, but the pituitaries of both groups showed a normal picture. As described by Griesbach and Purves (1945) and recently by Henegar and Higgins (1949), in the goitrogen-treated rat enlargement of thyroid cells is linked with an increase in numbers of pituitary basophil cells, indicating elevated thyrotropic activity. This correlation breaks down under the influence of A.A.F. Whereas the pituitary changes induced by the goitrogen disappeared when 2·5 μg. DL-thyroxine was injected for three weeks, the thyroids were far less affected. One half of the animals still had large goitres and, histologically, in all but one the epithelium was almost as high as in the rats which did not receive thyroxine.

No evidence for an increased demand for thyroxine or for higher sensitivity to thyrotropin was found in the animals treated with A.A.F. alone. Their thyroids and pituitaries were normal. When a thyroxine deficiency was induced in A.A.F. treated animals by surgical or chemical means, their pituitaries responded in the same way to thyroxine as did the animals without A.A.F. Similarly, the growth response of the female rats to thyroxine was independent of the presence of A.A.F. In the males, the pronounced liver damage prevented normal growth.

These facts show that the action of thyroxine on the pituitary was not inhibited by A.A.F. The problem can therefore be reduced to that of an anomalous behaviour of the thyroid epithelium. In the hands of Paschkis, Cantarow and Stasney (1948) L-thyroxine did not prevent the change from normal to high epithelium. In our experiments, physiological amounts of DL-thyroxine did not reduce a high cylindrical epithelium to normal. It must, however, be remembered that the hyperplastic thyroid epithelium of the A.A.F. treated animals was not completely refractory to thyroxine. By doubling the amount injected to 5·0 μg., inactive thyroids were obtained. We have no evidence suggesting that factors outside the thyroid were responsible for this phenomenon.

### SUMMARY.

Rats treated with acetamidofluorene and methylthiouracil responded to thyroxine in an anomalous manner.

With 2·5 μg. of DL-thyroxine injected for 21 days, the "thyroidectomy" pituitary was reconverted to its normal condition. This dose was insufficient to inactivate the thyroid which remained hyperplastic.

Under these conditions a dissociation existed between pituitary and thyroid.

The relative refractoriness of the thyroid cells must be attributed to the carcinogen acetamidofluorene.

The thyroid cells were inactivated by 5·0 μg. of DL-thyroxine in the presence acetamidofluorene.

We wish to thank Mr. T. H. Kennedy for supplying the solution of purified DL-thyroxine.

### REFERENCES.

Flaks, J.—(1948) *Brit. J. Cancer*, **2**, 395.

Griesbach, W. E., and Purves, H. D.—(1943) *Brit. J. exp. Path.*, **24**, 185.—(1945) *Ibid.*, **26**, 13.

Henegar, G. C., and Higgins, G. M.—(1949) *Amer. J. med. Sci.*, **218**, 251.

Hertz, R., and Tullner, W.—(1947) *J. nat. Cancer Inst.*, **8**, 121.

Martins, T.—(1933) *C. R. Soc. Biol., Paris*, **113**, 1275.

Paschkis, K. E., Cantarow, A., and Stasney, J.—(1948) *Cancer Res.*, **8**, 257.

Rawson, R. W., and Starr, P.—(1938) *Arch. intern. Med.*, **61**, 726.